# Dissecting postharvest chilling injuries in pome and stone fruit through integrated omics

**DOI:** 10.3389/fpls.2023.1272986

**Published:** 2024-01-03

**Authors:** Marta Rodrigues, Eduardo Javier Ordoñez-Trejo, Angela Rasori, Serena Varotto, Benedetto Ruperti, Claudio Bonghi

**Affiliations:** Department of Agronomy, Food, Natural Resources, Animals and Environment (DAFNAE), University of Padova, Legnaro, Italy

**Keywords:** cold storage, epigenomics, mealiness, *Rosaceae*, superficial scald

## Abstract

Lowering the storage temperature is an effective method to extend the postharvest and shelf life of fruits. Nevertheless, this technique often leads to physiological disorders, commonly known as chilling injuries. Apples and pears are susceptible to chilling injuries, among which superficial scald is the most economically relevant. Superficial scald is due to necrotic lesions of the first layers of hypodermis manifested through skin browning. In peaches and nectarines, chilling injuries are characterized by internal symptoms, such as mealiness. Fruits with these aesthetic or compositional/structural defects are not suitable for fresh consumption. Genetic variation is a key factor in determining fruit susceptibility to chilling injuries; however, physiological, or technical aspects such as harvest maturity and storage conditions also play a role. Multi-omics approaches have been used to provide an integrated explanation of chilling injury development. Metabolomics in pome fruits specifically targets the identification of ethylene, phenols, lipids, and oxidation products. Genomics and transcriptomics have revealed interesting connections with metabolomic datasets, pinpointing specific genes linked to cold stress, wax synthesis, farnesene metabolism, and the metabolic pathways of ascorbate and glutathione. When applied to *Prunus* species, these cutting-edge approaches have uncovered that the development of mealiness symptoms is linked to ethylene signaling, cell wall synthesis, lipid metabolism, cold stress genes, and increased DNA methylation levels. Emphasizing the findings from multi-omics studies, this review reports how the integration of omics datasets can provide new insights into understanding of chilling injury development. This new information is essential for successfully creating more resilient fruit varieties and developing novel postharvest strategies.

## 1 Introduction

Fruits and vegetables are among the most perishable foods throughout the food chain. Studies indicate that over 30% of produced fruits get wasted during harvest, storage, transport, and marketing (Boon, 2018). Besides the losses, fruits and vegetables can undergo a downgrade of some of their original characteristics throughout the supply chain, resulting in a reduction in their market value (Singh et al., 2007). Several factors can affect the original attributes of fruits. These can be mainly grouped into two categories that closely interact during the storage and handling of fruits: 1) endogenous factors, related to the intrinsic physiology of the fruit; and 2) exogenous factors, that can be of abiotic (e.g., temperature, humidity, atmosphere composition) or biotic (pathogens) nature. Ripening and senescence of fruits may occur within weeks or months depending on the genotype that affects fruit metabolism through its interactions with endogenous and exogenous factors. During ripening, climacteric fruits show an increase in ethylene production and respiration rate that significantly hasten their postharvest decay (Zenoni et al., 2023). These phenomena occur even if the fruit is detached at the end of its growth. Differently, non-climacteric fruits can ripe fully only if they remain attached to the plant (Zenoni et al., 2023). After ripening, the senescence process begins, leading to protein, lipid, and nucleic acid degradation at the cellular level, and more in general to cell dysfunction, and eventually cell death. During senescence, fruits may suffer from changes that can be detrimental to their appearance, texture, aroma, and nutritional value (Yang et al., 2014). Refrigeration is regularly exploited to reduce the overall metabolism, delay ripening, fruit respiration, and enzymatic activities, and, consequently, extend fruit shelf-life (Pott et al., 2020b). To enhance fruit preservation, cold storage (CS) is commonly combined with other post-harvest technologies including controlled atmosphere (CA), humidity control, treatment with ethylene blockers, ozone (O_3_), and other approaches to further delay fruit decay. While humidity control maintains the relative humidity at the highest levels tolerated, between 85 and 95%, CA always involves severe changes in atmosphere composition. The reduction of O_2_ and the increment of CO_2_ falls within ranges that can be very diverse depending on the specific fruit metabolism. In fact some fruits are very sensitive to small changes of these two gases (Fang and Wakisaka, 2021). Beyond the modifications of environmental conditions, the fruit postharvest behavior can be deeply altered by using chemicals. 1-methylcyclopropene (1-MCP) is a small molecule widely used to prevent ethylene-induced senescence. By binding irreversibly to ethylene receptors, 1-MCP effectively desensitizes the fruits, making them resistant to ethylene (Ekman et al., 2006). O_3_ can suppress diseases by surface sanitation and additionally by removing ethylene through its oxidation from the storage atmosphere (Ekman et al., 2006). All these techniques, together with low temperatures, significantly improve fruit storage and help decrease food waste.

CS of fruits, although improving their shelf-life, can cause physiological disorders known as chilling injuries (CIs). CIs, depending on the fruit, can lead to internal (IB) and external browning, mealiness, pitting and other manifestations of tissue necrosis (Pott et al., 2020b). It is common for these symptoms to be undetectable during CS. However, CIs become noticeable later, after the produce has been moved to the market, where the temperature is higher. Hence, the defects linked to CI typically result in customer dissatisfaction, thus determining a significant amount of fruit being wasted in the final stage of the fruit supply chain. In this view, the skin’s enzymatic browning is the primary factor affecting the consumer’s purchase (Zhang W. et al., 2023). Postharvest fruit peel browning is caused by polyphenol oxidase (PPO) activity. PPO oxidizes phenols to o-quinones in the presence of O_2_ or H_2_O_2_, originating brown-colored polymers (Franck et al., 2007). Both the mealy texture and surface pitting are caused by a significant change in the turnover of all components of the cell wall network. However, it has been recognized that the main cold defects at the cell wall level are primarily caused by the reduced solubilization and depolymerization of the pectin (Fruk et al., 2014; Stanley et al., 2023).

All these abnormalities can be linked to specific cellular dysfunctions as summarized by Albornoz et al. (2022). These authors described a possible roadmap for CI development in different plant tissues. For fleshy fruit, Albornoz’s scheme has been developed on tomatoes, the model species for fleshy fruits as those harbored by *Rosaceae* species. Briefly, at the cellular level, CS temperature induces changes in membrane composition and fluidity by the enhanced activity of phospholipases (PLs) and lipoxygenases (LOXs), the main enzymes responsible for the hydrolysis of phospholipids (Aghdam et al., 2014). Membrane peroxidation can be also exacerbated by reactive oxygen species (ROS) that can reach harmful levels during CS, caused by their inefficient scavenging (Imahori et al., 2016). The alteration of membrane structure together with the reorganization of the cytoskeleton, and disturbances in the proper functioning of calcium channels leads to uncontrolled ions, mainly calcium, influx into the cytoplasm (McAinsh and Pittman, 2009). Calcium induces mitogen-activated protein kinases (MAPKs) and Ca^2+^/calmodulin (CaM)-dependent kinases for amplifying cold signals (Aghdam et al., 2012; Tang et al., 2021). MAPKs are also involved in the signal transduction of cold-related changes in hormone levels [in particular, ethylene bursts, Albornoz et al. (2019)] resulting in the activation of cold-related pathways, such as the ROS scavenging and the unbalance of energy sources (Shu et al., 2022). All these events determine changes in the transcription of cold-related genes. Gene transcription during CS is regulated at multiple layers such as the binding of transcription factors to cold-regulative elements located in the promoter [e.g. C‐repeat-binding factors, CBFs, Zhang et al. (2004); Albornoz et al. (2019)], the level of methylation in the promoter and gene body, the acetylation status of histones in the nucleosomes (Aiese Cigliano et al., 2022). At the post-transcriptional layer the transcript level of cold-responsive genes can be modulated by microRNAs (Aiese Cigliano et al., 2022). This roadmap provides a promising blueprint for organizing information coming from studies on various refrigerated fleshy fruits, for which in certain cases contrasting results have been produced (Zhang et al., 2021). Contradictory findings emerge when examining the impact on the development of fruit CI of some postharvest treatments, such as ethylene and 1-MCP (Zhang et al., 2021). In addition, when applying postharvest technologies, it is also important to consider key factors, such as the time and intensity of the treatment. The intensity and the duration of the cold stress are key elements and Albornoz et al. (2022) suggested that if the CS is mild and temporary, CI may not occur and fruit metabolism may resume after rewarming or if intermittent warming is applied to break the cold period (Watkins et al., 1995; Cainelli and Ruperti, 2019). On this view, the combination of omics (proteomics, metabolomics, transcriptomics, etc.), can be a very powerful tool to understand how CI can impact quality traits and to model the regulatory aspects of CI induction and symptoms development. These tools also help understand the reorganization of fruit metabolism because of abiotic stress caused by postharvest storage conditions and its influence on the metabolites responsible for aroma, taste, and nutritional quality, directly impacting consumers choices. The identification of biomarkers for all these changes could be used by the fruit industry to determine the variations in terms of quality during CI (Pott et al., 2020a; Pott et al., 2020b). Following this idea, Albornoz and co-workers (2019) proposed some biomarkers (respiration and ethylene rates, malondialdehyde, and starch content) strictly related to postharvest CI in cherry tomatoes by integrating omics data.

Although molecular mechanisms activated during CI have been investigated, there is no recent review that covers the latest multi-omics studies for fleshy fruit of Rosaceae species. The present work aims to review and organize the multi-omics data, focusing on the pome (*Maloideae*) and stone (*Prunoideae*) fruits, as they represent a largely significant part of cultivated fruits that suffer from CI during CS, using as a blueprint the roadmap developed by Albornoz and co-workers (2022). To effectively implement the model, we have also incorporated information derived from chemical treatments aimed at alleviating CI. This inclusion is based on the notion that chemicals have the potential to modify postharvest phenotypes, thus allowing the establishment of coarse links between modulated genes and the observed alterations. This approach is a realistic possibility waiting for the application of gene editing for reducing postharvest losses (Shipman et al., 2021) in crop species recalcitrant to transformation as the stone fruit trees (Ricci et al., 2020). Moreover, we discuss some data integration strategies able to help in the organization, analysis, and correlation of postharvest big dataset.

## 2 Integrated omics for studying chilling injury development and its mitigation

### 2.1 Maloideae

The *Maloideae* subfamily contains up to 1,000 species, of which the most emblematic are apples (*Malus domestica* Borkh.) and pears (*Pyrus communis* L.) (Phipps et al., 1991). Although apples and pears are climacteric fruits, in which ripening is tightly associated with a peak in respiration and a simultaneous burst of ethylene leading to a rapid ripening and decay, they can be stored for long periods, resorting to low temperature and CA technologies (Kupferman, 2003; Payasi and Sanwal, 2010). As stated by Kupferman (2003), apple storage can go up to 12 months and that of pear up to 9 months, depending on the variety. However, during long storage at low temperatures, pome fruits can suffer from disorders arising from biochemical changes occurring consequently to prolonged exposure to low non-freezing temperatures.

#### 2.1.1 CI in apples and pears

CI in apple and pear starts as a physiological syndrome finally leading to tissue decay and develops into increased susceptibility to pathogens. Apples and pears disorders may involve the skin and the sub-epidermal cortex layers of cells (superficial scald) but can also reach the outermost layers of cortical tissues (e.g. soft scald and bitter pit), or involve browning of entire internal portions of the pulp (internal breakdown) or of the fruit core (brown core) (Gapper et al., 2023; Prange and Wright, 2023). Among these, superficial scald has received considerable efforts of research activity. Previous studies hypothesized that scald is a programmed disorder, triggered by epigenetic modifications of specific genes when fruits are subjected to oxidative stress (Karagiannis et al., 2018). Investigations with Granny Smith apples verified that peel tissue necrosis, a symptom of superficial scald, is an actively programmed process by which metabolic transitions to cell death are regulated, in a way that may be similar to a hypersensitive response to pathogens or programmed cell death (Gapper et al., 2017). Decrease in photosynthesis, emphasizing by changes in expression of chloroplast genes, is one of the earliest hallmarks of plant cell death (Ambastha et al., 2015). During the development of CI symptoms in Granny Smith skin tissues, as they became necrotic, there was a noticeable decrease in the chlorophyll content and the expression of photosystem2 genes (Gapper et al., 2017). However, it is worthy to note that these events have only been reported for apple green varieties that retain their green color both at harvest and during postharvest.

The integration of “omics” approaches with biochemical parameters of CI allow to highlight certain metabolic pathways that have been correlated with CI, such as the farnesene (FAR) pathway (Karagiannis et al., 2018; Lindo-García et al., 2020; Marc et al., 2020; He et al., 2022; Vittani et al., 2023), the ethylene pathway (Wei et al., 2019; Karagiannis et al., 2020; Koushesh Saba and Watkins, 2020), the phenylpropanoid pathway (Gapper et al., 2017; Koushesh Saba and Watkins, 2020; Marc et al., 2020; Busatto et al., 2021; He et al., 2022; Vittani et al., 2023; Zhang C. et al., 2023) and the fatty acid metabolism pathway (Giné-Bordonaba et al., 2020; Busatto et al., 2021; Vittani et al., 2023; Zhang C. et al., 2023).

FAR is a sesquiterpenoid produced by plants and animals. One of the most common forms of this compound in plants is α-FAR, highly correlated with ethylene and known for its increase during apple ripening. Despite its abundance, the biological function of α-FAR is rather uncertain (Souleyre et al., 2019). Many studies have linked the FAR pathway with CI. According to Vittani et al. (2023), apples (Ladina and Granny Smith varieties) stored at low temperature were enriched in the pathway of farnesyl diphosphate, an α-FAR precursor. Additionally, other studies highlighted the upregulation of genes related to FAR in pears susceptible to CI after CS (Lindo-García et al., 2020; He et al., 2022). On the other hand, Karagiannis et al. (2020) found a downregulation of farnesyl diphosphate synthase 2 (*FPS2*) gene in an apple genotype tolerant to CI. Previously, this author had found an induction of genes involved in the generation of α-FAR in Granny Smith apples, the oxidation of which has been linked to the development of superficial scald disorder (Lurie and Watkins, 2012; Karagiannis et al., 2018). Contrastingly, Marc et al. (2020) conducted an experiment with apples that led to the conclusion that FAR profiles cannot fully explain the lack of symptoms after acclimation treatments. This study suggested that tolerance to CI is more dependent on phenylalanine ammonia-lyase rather than on FAR (Marc et al., 2020). This result supports a vision in which the role of FAR in superficial scald must be revised as already suggested by Lurie and Watkins (2012).

Ethylene has been strongly correlated with the development of CI syndromes in apples and pears. According to Karagiannis et al. (2020), CS of apples reduced the expression of 1-aminocyclopropane-1-carboxylic acid oxidase (ACO), responsible for the production of ethylene. Furthermore, Koushesh Saba and Watkins (2020) measured the internal levels of ethylene and determined that when this parameter was low, a higher incidence of flesh browning was observed. As a result, they hypothesized that a low concentration of ethylene is required to preserve homeostasis and stress tolerance of Empire apples, the opposite of what was detected in Granny Smith at the onset of superficial scald (Koushesh Saba and Watkins, 2020). Moreover, during shelf life, Empire apples that had been kept at 0.5°C rapidly increased internal ethylene levels, probably because of the accumulation of 1-aminocyclopropane-1-carboxylate (ACC), an ethylene precursor (Koushesh Saba and Watkins, 2020). Other studies have implicated ethylene in the CI of pears. Wei et al. (2019) treated Huangguan pear with ethylene and determined that it inhibited the accumulation of ROS and consequently prevented the development of browning syndromes. The authors proposed that high levels of ethylene might help to prevent CI in pear and should be considered as a mitigation strategy (Wei et al., 2019).

As far as secondary metabolism is concerned, phenylpropanoids, a class of compounds produced from the amino acid phenylalanine acting as signal molecules both in plant development and defense (Dixon et al., 2002), have been claimed to play a role in CI. Vittani et al. (2023) determined by transcriptomic analysis that CI susceptible apples (Ladina and Granny Smith) stored at low temperature were enriched for the expression of genes of the biosynthetic pathway of phenylpropanoids. The same was observed for susceptible pears, where genes participating in the phenylpropanoid pathway were upregulated upon setting of CI (He et al., 2022). A multiplicity of studies has identified PPO as a major enzyme impacting CI. PPO oxidizes phenolic compounds to quinones, in a process known as enzymatic browning (Yoruk and Marshall, 2003). The upregulation of the gene encoding PPO (Gapper et al., 2017) and the higher activity of PPO (Koushesh Saba and Watkins, 2020), during the shelf-life of 1-MCP treated fruit, was determined to be associated with the development of CI in apples. However, Marc et al. (2020) found that PPO by itself was not able to justify the observed syndromes in Granny Smith after CS. Additionally, He et al. (2022) determined that Wujiuxiang variety of pears (susceptible to CI) showed low expression of PPO when IB was set. Contrastingly, according to Busatto et al. (2021), tolerant pear Conference showed low expression of PPO and had higher quantities of flavonoids and other phenolic compounds compared to the susceptible pears. Moreover, in this study, it was determined that the tolerant pear Conference had high levels of chlorogenic acid (CGA). CGA is a phenolic acid associated with the delay of senescence (Cheng et al., 2020). Likewise, susceptible pears have been found to have low levels of CGA (He et al., 2022). Furthermore, Zhang C. et al. (2023) demonstrated that flavonoids (particularly kaempferol-3-O-sambubioside and luteolin-7-O-(6′-malonyl) glucoside) were more accumulated in tolerant pears and were responsible for the main differences observed between tolerant and susceptible phenotypes. Additionally, metabolome analyses pointed out high concentrations of catechin, epicatechin, and procyanidin in apples showing tolerance to CI (Vittani et al., 2023). These compounds are polyphenols able to prevent oxidation, and their presence has been associated with a decrease in oxidative stress (Fraga and Oteiza, 2011).

Several studies have pointed out the fact that peroxidases (POX), catalyzers of the oxidation of multiple compounds, might be connected to CI. Karagiannis et al. (2020) and Koushesh Saba and Watkins (2020) detected an increase in POX activity in apples suffering from CI. The same was observed with the pear Flor d’Hivern, susceptible to CI, which after the development of scald also increased the activity of POX. The authors suggested that this may be due to POX’s action on membrane peroxidation (Lindo-García et al., 2020).

Lately, CI has been associated with fatty acid metabolism. Vittani et al. (2023) detected an enrichment of genes participating in fatty acid metabolism, particularly related to jasmonates. These authors hypothesized that superficial scald may be regulated differently according to the variety of apples and such regulation may involve jasmonates. Additionally, this study detected lower content of fatty acids, particularly palmitic, oleic, linoleic, and linolenic acids, after CI installment. The lipid profile of Ladina apples, susceptible to CI, changed after the CI set, disfavoring the accumulation of very long-chain fatty acids (VLCFAs). This discovery supported the hypothesis that the membrane integrity can change faster in apples of the susceptible varieties, causing CI (Vittani et al., 2023). Other studies detected an increased amount of VLCFAs in Conference pears (tolerant) (Busatto et al., 2021) and of free fatty acids (namely eicosanoid acid, elaidic acid, and 11-octadecanoic acid) and VLCFAs in tolerant pears (Zhang C. et al., 2023).

Other metabolic pathways were identified as participating in CI, namely pathways related to defense mechanisms. Glutathione S-Transferase (GST), besides its role in cell proliferation, apoptosis, growth, and development, enhances plant tolerance to different abiotic stresses (Hasanuzzaman et al., 2017). The GST gene was found to be transcriptionally upregulated in the presence of superficial scald. The authors of this study theorized that GST upregulation could be associated with the detoxification against reactive aldehydes or, alternatively, GSH could be produced to strengthen the control of ROS (Karagiannis et al., 2020). An upregulation of the gene encoding glutathione reductase was observed in tolerant apples (Vittani et al., 2023). Glutathione reductase has a major role in the conversion of glutathione disulfide (GSSG) into glutathione (GSH), preventing oxidative stress (Deponte, 2013). Indeed, the entire network of genes encoding enzymes involved in the control of ROS levels is transcriptionally rewired during scald induction, indicating that the balanced action of ascorbate and GSH recycling enzymes plays a role in this process (Zermiani et al., 2015). Additionally, Karagiannis et al. (2020) detected an upregulation of serine in tolerant apples that might indicate an activation of antioxidant defenses and an accumulation of several amino acids was observed which may play an important role in CI tolerance. The authors suggested that an adaptive strategy to prevent scald might be nucleo-cytoplasmic transport of proteins, without *de novo* synthesis, which would allow faster responses to changes in temperature (Karagiannis et al., 2020).

#### 2.1.2 CI mitigation strategies in apples and pears as tools to dissect metabolomics and transcriptomic links to CI

The development of browning disorders can be influenced by several external factors as climatic conditions and level of maturity at harvest, but also by specific characteristics related to the variety of fruits (fruit size, level of crop load, etc.) (Sidhu et al., 2023). The use of resistant cultivars is the first approach that can be used to avoid CI (Gapper et al., 2023). Apple trees that possess resistance to cold tend to generate apples that can easily be stored at low temperatures without the occurrence of browning syndromes (Xu et al., 2023). Currently, there are varieties of apples identified as susceptible to CS stress, such as Delicious, Fuji, Granny Smith, Ladina, and Honeycrisp (Leisso et al., 2016; Vittani et al., 2023). Likewise, Huangguan, Nanguo, and Blanquilla pears are identified as being susceptible to CS (Busatto et al., 2021; Cheng et al., 2022; Yang et al., 2023). In the susceptible variety Honeycrisp two quantitative trait loci (QTLs) physiologically related to superficial scald and soggy breakdown have been identified (Howard et al., 2018). Although fine mapping of these two QTLs was limited by the size of the population, the authors proposed *MdAAT1* as candidate gene, which codes for an alcohol acyl-transferase that catalyzes the transacylation from acyl-CoA to alcohol. The suppression of transacylation led to increased levels of hexanol and other alcohols (Souleyre et al., 2014). The *MdAAT1* function is supported by the increased risk of soft scald formation in ‘Honeycrisp’ apple, after hexanol injection or in the presence of a high level of this alcohol (Leisso et al., 2016). A comparison between pear cultivars showing different susceptibility to superficial scald pointed out that the ascorbate level rather than the total level of antioxidants is significantly related to the tolerance. Therefore the attention on selecting more tolerant cultivars has been driven by this piece of evidence (Larrigaudière et al., 2016). Current efforts toward the identification of tolerant cultivars are focused on the development of molecular markers for future marker-assisted selection programs.

Although opting for resistant genotypes is the most efficient way of mitigating CI, there is a heavy limitation to this strategy: the success of newly developed resistant apple varieties is largely dependent on their fruit quality, as it was observed for those resistant to postharvest disease (Baumgartner et al., 2015). Considering this limitation, currently other strategies are being employed on susceptible varieties. A multiplicity of studies has attempted to identify treatments that can be performed before or during storage and that might reduce CI physiological disorders. **Table 1** reports recent studies in the *Maloideae* family that used several omics approaches and were dedicated to understanding how CI disorders are characterized and how they can be avoided.

**Table 1:** Recent studies devoted to apple and pear chilling injury.

Fruit (Variety)	Reference	Topic	Omics studied	Omic data analysis
**Apples (Granny Smith)**	Karagiannis et al. (2018)	Effect of 1-MCP and DPA on apples stored at 0 °C	Proteomics, Transcriptomics, Metabolomics. Epigenomics	DAP, DAM, DEG, DMR, GO, KEGG pathways,
**Apples (Granny Smith and Ladina)**	Vittani et al. (2023)	Effect of low T conjugated with 1-MCP and low O_2_	Transcriptomics, Metabolomics	DAM, DEG, GO, KEGG pathways, VD, CbI
**Apples (Empire)**	Koushesh Saba and Watkins (2020)	Effect of 1-MCP and low T	Proteomics, Transcriptomics, Metabolomics	DAM, DEG
**Apples (Granny Smith)**	Karagiannis et al. (2020)	Effect of 1-MCP and O_3_ conjugated with low T	Proteomics, Transcriptomics, Metabolomics	DAM, DAP, DEG, MapMan, NA, Venn
**Apples (Granny Smith)**	Gapper et al. (2017)	Effect of 1-MCP and DPA on apples stored at 1°C	Metabolomics, Transcriptomics	DAM, DEG, MBPLS-DA, VIP scores, NA, Mapman, ORA
**Pears (Wujiuxiang and Yali)**	He et al. (2022)	Effect of storage at 0 °C	Metabolomics, Transcriptomics	DAM, DEG
**Pears (Blanquilla and Conference)**	Busatto et al. (2021)	Effect of low T conjugated with 1-MCP and LOV	Metabolomics, Transcriptomics	DEG, DAM,
**Pears (Chili)**	Zhang W. et al. (2023)	Bagging (PE and NWF) and low T storage	Transcriptomics, Metabolomics	DEG, DAM, Venn, Correlation analysis
**Pears (Huangguan)**	Wei et al. (2019)	Effect of ethylene with 0 °C storage	Transcriptomics, Metabolomics	DAM, DEG
**Pears (Blanquilla and Flor d’Hivern)**	Lindo-García et al. (2020)	Effect of ethylene with -0.5 °C storage	Transcriptomics, Metabolomics	DAM, DEG

In this table, the following acronyms were used: 1-MCP, 1-methylcyclopropene; CbI, correlation based interactome; DAM, differentially accumulated metabolite; DAP, differentially accumulated proteins; DEG, differentially expressed genes; DPA, diphenylamine; GO, Gene Ontology; KEGG, Kyoto Encyclopedia of Genes and Genomes; LOV, lovastatin; MapMan, , MapMan functional enrichment analysis; MBPLS-DA, multi-block partial least squares discriminate analysis; NA, network analysis; NWF, non-woven fabric; ORA, overexpression analysis; PE, polyethylene; T, temperature; Venn, venn diagram; VIP score, variables' importance in projection.

The chemical and physical strategies used commercially to prevent the occurrence of CI have also been exploited by researchers to identify the underlying molecular and metabolic factors. As mentioned in the previous section, ethylene is a major contributor to CI development (Wei et al., 2019; Karagiannis et al., 2020; Koushesh Saba and Watkins, 2020). 1-MCP has been widely studied for its ethylene inhibitory action and is known to significantly reduce or fully prevent the superficial scald of Granny Smith apples (Lurie and Watkins, 2012). 1- MCP treatment results in a complete rewiring of the gene network involved in the control of ROS homeostasis through a balanced ratio between the production and metabolism of these molecules. Thus, 1-MCP impacts the ability of fruits to cope with oxidative stress and may act at least in part by preventing oxidative decay and cell death (Zermiani et al., 2015). Karagiannis et al. (2018) evidenced the fact that 1-MCP promoted isoleucine biosynthesis, an event that has been associated with CI mitigation (Tanou et al., 2017). Additionally, the application of 1-MCP also led to a reduction in the expression of α-FAR synthase, which has been linked to a decrease in the occurrence of CIs in the treated apples (Karagiannis et al., 2018). However, it is more likely that the impact of 1-MCP on FAR levels is indirect. Indeed, Lurie and Watkins (2012) reported that 1-MCP could regulate genes involved in cutin, suberine, and wax biosynthesis. Consequently, the changes in wax composition could affect the accumulation and diffusion of volatiles, such as α-FAR.

Koushesh Saba and Watkins (2020) also investigated the effect of 1-MCP and low-temperature storage of Empire apples on the development of flesh browning disorder. By measuring internal ethylene concentration (IEC), they could show an inhibition of ethylene biosynthesis in 1-MCP treated samples, but also in samples stored at 0.5 °C. A similar study conducted by Gapper et al. (2017) surveyed the metabolomics and transcriptomics of 1-MCP treatments in Granny Smith, after long time storage at low temperature (1°C). The comparison of metabolome and transcriptome has been exploited to dissect the preventive effects on superficial scald exerted by physical (low oxygen) and chemical (1-MCP) methods in varieties characterized by different susceptibility to developing CI. For this goal Vittani et al. (2023) conducted a study on Granny Smith (considered a medium susceptible variety to CI) and Ladina (a new variety highly susceptible to CI), demonstrating that Granny Smith apples treated with 1-MCP or with low oxygen were not susceptible to CI. This investigation concluded that 1-MCP controlled superficial scald by reprogramming fatty acid composition (Vittani et al., 2023). Karagiannis et al. (2020) investigated the combined effect of 1-MCP and O_3_ in Granny Smith apples stored at 0°C. While, as expected, 1-MCP prevented superficial scald, O_3_ treatment induced this disorder and the combined effect of both treatments (1-MCP and O_3_) resulted in the prevention of the CI, confirming that superficial scald is an ethylene-dependent CI and that 1-MPC is very efficient in the reversion of O_3_ effects. The ability of 1-MCP to counteract O_3_-induced superficial scald is not dependent on blocking ethylene biosynthesis or its function. This is because O_3_ does not impact the production of ethylene or the activities of enzymes like ACS and ACO. The contrasting impact of 1-MCP and O_3_ appears to be closely tied to a distinct activation of proteins responsible for regulating gene expression at the posttranscriptional and translational levels. Generally, CS caused a reduction of several amino acids (e.g., aspartic acid, serine, valine), however, threonine and valine were present at higher levels after the application of 1-MCP, leading to the conclusion that the ethylene blocker reprograms the branched-chain amino acids (BBCAs) biosynthesis through the induction of their biosynthetic genes (Karagiannis et al., 2020). This result agrees with data obtained in peach fruit showing that BCAAs’ high accumulation confers tolerance against CIs (Tanou et al., 2017). Similar studies performed on pears showed partially overlapping or even contrasting evidence compared to those found in apples. This could imply that different regulatory mechanisms may exist within the same family of fruits. Two studies (Giné-Bordonaba et al., 2020; Busatto et al., 2021) investigated the effect of 1-MCP and lovastatin (inhibitor of the mevalonate pathway leading to α-FAR biosynthesis) treatments on Blanquilla and Conference pears stored at 0.5°C. Conference pears showed very little effect of low temperature storage while Blanquilla presented a significant amount of superficial scald. However, the treatments were effective in preventing the set-up of CI. Busatto et al. (2021) hypothesized that the increased amount of VLCFAs detected after treatment was possibly linked to the tolerance mechanism.

Besides 1-MCP, also diphenylamine (DPA) is a known chemical responsible for the reduction or even prevention of superficial scald (Lurie and Watkins, 2012), due to its antioxidant properties. Karagiannis et al. (2018) studied the effects of DPA on Granny Smith apples stored at 0°C. Superficial scald was significantly reduced by the action of DPA. DPA upregulated the level of serine/threonine-protein phosphatase 2B regulatory subunit, a compound that participates in signal transduction under abiotic stress. Moreover, it induced UDP-xylose and UDP-arabinose. The authors hypothesized that DPA interfered with the nucleotide sugar interconversion pathways and that ultimately led to an increase in nucleotide sugars within the cell wall, keeping its flexibility after cold exposure (Karagiannis et al., 2018). Gapper et al. (2017) also used DPA to prevent CI injury in apples. The authors determined that during CS of apples, the metabolism (enhancement of volatile biosynthesis and diminishment of malic acid) of DPA-treated and control samples, unlike 1-MCP treated samples, was regulated along the ripening process. On the other hand, methanol and methyl ester production occurred in these samples only during symptom development (Gapper et al., 2017).

A recent study (Zhang C. et al., 2023) investigated the possibility of using pre-harvest treatments on pears to prevent CI. This work focused on the usage of bags (polyethylene, PE, and non-woven fabric, NWF) to cover Chili pears during fruit development to mitigate CI after 4°C storage. PE-bagged pears resulted in superficial scald-sensitive fruits while NWF prevented this disorder. In asymptomatic fruits, the cuticular wax structure was more complete and its concentration was greater. Cuticular wax was mainly composed of VLCFAs, catalyzed by β-Ketoacyl CoA synthase (Zhang C. et al., 2023).

Lindo-García et al. (2020) further studied the effect of ethylene in two pear cultivars Blanquilla (a variety that produces ethylene) and Flor d’Hivern (which does not produce ethylene), with different harvest dates followed by -0.5°C storage. Both varieties were susceptible to CI but while Blanquilla was influenced by the harvesting date and only showed symptoms during shelf life, Flor d’Hivern was not influenced by the maturity state and showed symptoms already during storage. Blanquilla showed upregulation of the FAR synthase gene when the fruits started the ripening process on the tree. Apparently, Flor d’Hivern had, CI independently from ethylene. This cultivar developed scald associated with POX and lysyl oxidase activities, probably due to their action in membrane peroxidation. This reaction together with a reduced content of sorbitol might explain the development of the disorder in this cultivar (Lindo-García et al., 2020).

Some of the pathways identified in the previously mentioned treatment might be used to individuate genes responsible for conferring CI resistance to fruits. The use of QTL analysis, currently under development, could be of major importance in the near future to early detect which varieties of apples and pears might be tolerant to CI (Gapper et al., 2023).

**Figure 1:**
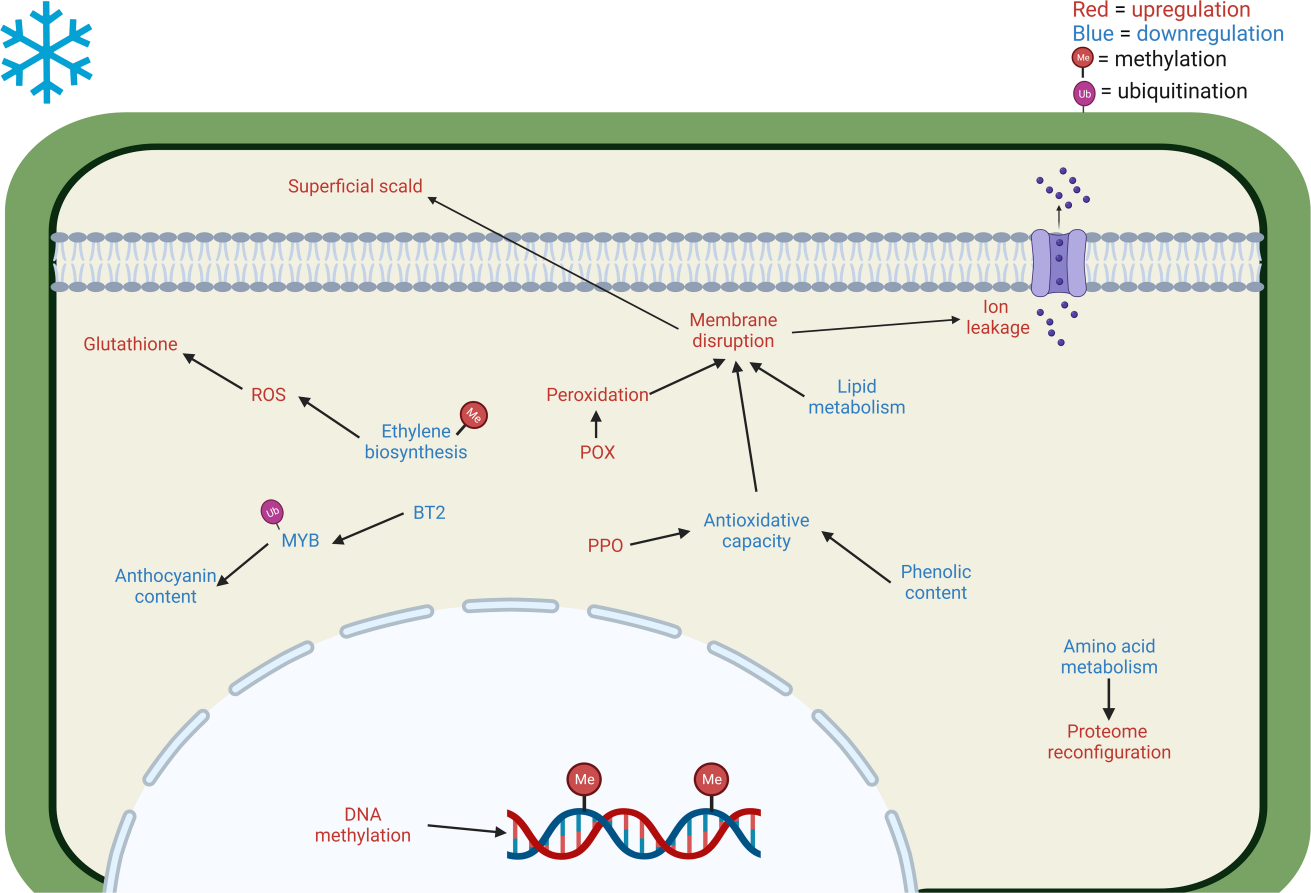
Main cellular and metabolic responses in *Maloideae* fruits to cold stress revealed through multi-omics approaches. The blue letter coloring implies a downregulation of the pathway/decrease in the accumulation of the compound. On the other hand, red letter coloring represents an upregulation/increase in the accumulation of the compound. CI increases DNA methylation (Karagiannis et al., 2018) and decreases amino acid biosynthesis such as aspartic acid, serine, and valine (Karagiannis et al., 2020), reconfiguring the proteome. The decrease of phenols (Vittani et al., 2023), together with an increase in PPO (Gapper et al., 2017; Koushesh Saba and Watkins, 2020) reduce the antioxidative capacity, leading to membrane disruption. Moreover, the membrane is further affected by the decrease of lipid metabolism, particularly palmitic, oleic, linoleic, and linolenic acids, VLCFAs, and free fatty acids (Busatto et al., 2021; Vittani et al., 2023) and increase of peroxidation due to the upregulation of POX (Karagiannis et al., 2020; Lindo-García et al., 2020). The membrane disturbance causes ion leakage and superficial scald. Furthermore, ethylene biosynthesis is affected by the downregulation of ACO (Karagiannis et al., 2020), causing the increment of ROS (Wei et al., 2019) and consequently the upregulation of glutathione (Karagiannis et al., 2020).

All previous information (see 2.1.1 and 2.2.1. paragraphs) can be used to adapt the model proposed by Albornoz et al. (2022) to cold stored pome fruits (**Figure 1**) . However, the model of CI induction and development for these fruits remains a difficult task and should necessarily consider the fact that pear and apple fruits (or even different varieties of the same type of fruit) may differ significantly or even display opposite regulatory features. There has been significant progress toward the identification of the transcriptional, proteomic, and metabolic changes accompanying the induction and development of CI in apples and pears by “omics” approaches. On the contrary, very little is currently known about the epigenetic regulation of these events in pome fruits, and, to our knowledge, there are no multi-omics studies that specifically evaluated the epigenetic states of genes in apples and pears subject to CS. Indeed, epigenetic changes occurring during storage might explain the induction of CIs in pome fruits. Gapper et al. (2013) speculated that the environmentally induced impacts of CI in apples might be related to the control of ethylene biosynthesis through DNA methylation. This study suggested that ACC synthase 1 (*ACS1*) might be one of the genes involved in this regulation, but it is likely that other genes may also be under the control of epigenetic mechanisms (Gapper et al., 2013). Karagiannis et al. (2018) reported that the preventive action of DPA and 1-MCP on the induction and development of superficial scald in Granny smith apples is associated with a stimulation of methylation events. Nevertheless, systematic studies on the epigenetic regulation of CI in apples and pears are lacking and this field of research is still in its infancy.

### 2.2 *Prunoidea*e

The *Prunoideae* is a subfamily of *Rosaceae* and contains economically important species from the *Prunus* genus such as peaches, plums, apricots, almonds, and cherries. They are characterized by having a drupe (or stone fruit) as a fruit. Stone fruits have a thin exocarp, a fleshy mesocarp, and a lignified endocarp that surrounds the seed. *Prunus* fruits are mostly climacteric and have a short ripening and shelf-life period at ambient temperature, therefore, CS is the main treatment to extend their postharvest life.

#### 2.2.1 CI in *Prunus* fruits

CS triggers low-temperature disorders in *Prunus* fruits, limiting their storage life. The main CIs in *Prunus* are mealy or wooly texture (mealiness or wooliness), flesh browning, flesh bleeding/reddening, flesh translucency, flavor loss, and reduced juiciness and softening capacity (leatheriness) (Crisosto et al., 2004; Lurie and Crisosto, 2005; Stanley et al., 2013). The development of these CIs is a quantitative trait with complex genetic regulation and it is influenced by the preharvest history of the fruits (Lurie and Crisosto, 2005). QTLs related to CIs in peaches and nectarines have been mapped in linkage groups 1, 4, and 5, however, QTLs for specific CIs usually overlap and contain many candidate genes, which are generally suggestive (Lurie, 2021). Genomic studies in *Prunus persica* have been limited mainly due to the reduced number of individuals produced in the F2 population and by the simultaneous development of multiple CIs (Lurie, 2021). Additionally, the discovery of numerous QTL have been performed before the development of novel genomic resources and technologies (Lurie, 2021). As an alternative to genomic studies, the transcriptomic comparison between genotypes with different cold sensitivity and the comparison between physical or chemical CI alleviation treatments have been used to identify genes involved in CIs. Studying the function of the candidate genes inside these QTLs would connect them to the development of specific CIs. However, the lack of an efficient genetic transformation system prevents biotechnological approaches for functional genomic studies (Ricci et al., 2020). Due to the complex genetic regulation and inheritance of CI tolerance-related genes, breeding programs can rely on selecting genotypes with high bioactive compound contents that are known to contribute to low CI incidence i.e. sugar and flavonoid content (Rodrigues et al., 2022).

Recent reviews have discussed the use and impact of omics on peach and nectarine CI research discussing each omics layer individually (Lurie, 2021; Lurie, 2022). In this review, we discuss the use of multi-omics approaches in *Prunus* CI research, also considering epigenetic regulation. **Table 2** mentions several recent studies that were dedicated to understanding how these disorders were characterized and how they could be alleviated. The role of epigenetics in stone fruit CI development was assessed by Rothkegel et al. (2021) and Zhu et al. (2021), using transcriptomics and DNA methylation in nectarines (Venus) and peaches (Zhonghua**s**houtao), respectively, during CS. These authors found that methylation levels were higher in fruits with CIs, particularly in the CHH context. Rothkegel et al. (2021) associated higher methylation levels with mealiness; meanwhile Zhu et al. (2021) found that methylation levels correlated positively with browning index (BI). The latter authors also highlighted the role of demethylation in temperature-dependent anthocyanin accumulation (Zhu et al., 2020). At 16 °C, no CI development was reported, however, it was the only temperature at which anthocyanin accumulation was observed in an originally white-fleshed cultivar during its shelf-life (Zhu et al., 2020). Therefore, CS induces global changes in peach fruit DNA methylation, which vary according to storage conditions.

**Table 2:** Recent studies devoted to *Prunus* chilling injury.

Fruit (Variety)	Reference	Topic	Omics studied	Omic data analysis
Nectarine (Venus)	Rothkegel et al. (2021)	Effect of storage at 0 °C	Transcriptomics, Epigenomics	DEG, DMR, GO
Peach (Zhonghuashoutao)	Zhu et al. (2020); Zhu et al. (2021)	Effect of storage at 0, 5, 8, 12 and 16 °C	Transcriptomics, Epigenomics	DMR, DEG, GO, KEGG pathways, WGCNA, AA
Peach (Flaminia and Red Haven)	(Brizzolara et al., 2020)	Effect of storage at 0.5 and 5.5 °C	Transcriptomics, Metabolomics	ANOVA, EA
Peach (Hujingmilu)	Duan et al. (2022)	Effect of MeJA on storage at 0°C	Transcriptomics, Epigenomics, Metabolomics	DEG, DMR, GO, Venn
Peach (Spring Lady)	Monti et al. (2019)	Effect of storage at 0 °C	Proteomics, Metabolomics	DAP, GO, PNA
Peach (June Gold)	Tanou et al. (2017)	Effect CS and PC at different maturity stages	Transcroptomics, Metabolomics, Proteomics	ANOVA, Venn, DEG, DAP, DAM
Plum (Friar)	Xu et al. (2022)	Effect of melatonin on storage at 0 °C	Metabolomics, Transcriptomics	DEG, GO, KEGG, Venn, DAM, Phylogenetic analysis
Peach (Hujingmilu)	Wang et al. (2017)	Effect of LTC on cold storage	Metabolomics, Transcriptomics	DEG, MapMan, WGCNA, HCA, GO, Venn, DAM, MRT
Peach (Yuhua No.2)	Wang L. et al. (2021)	Effect of hot water treatment in stored peaches at 0 °C	Metabolomics, Transcriptomics	DEG, ANOVA, MRT, DAM, KEGG, OPLS-DA,
Peach (Jinqiuhongmi)	Zhao et al. (2021b)	The effect of JA and SA on cold storage	Metabolomics, Transcriptomics	DEG, MRT, DAM
Peach (Zhonghuashoutao)	(Zhu et al., 2019)	The effect of exogenous ethylene on cold storage	Metabolomics, Transcriptomics	DEG, GO, WGCNA, HCA, DAM, Venn

In this table, the following acronyms were used: AA, association analysis; DAM, differentially accumulated metabolites; DAP, differentially accumulated proteins; DEG, differentially expressed genes; DMR, differentially methylated regions; EA, expression analysis; GO, gene ontology; HCA, hierarchical clustering analysis; JA, jasmonic acid; KEGG, Kyoto Encyclopedia of Genes and Genomes; LTC, low temperature conditioning; MapMan,, MapMan functional enrichment analysis; MeJA, methyl jasmonates; OPLS-DA, Orthogonal Partial Least Squares Discrimination Analysis; PNA, protein network analysis; SA, salicylic acid; T, temperature; Venn, venn diagram; and WGCNA, weighted gene coexpression network analysis.

To understand whether the changes in DNA methylation were correlated to the expression of genes coding for peach methyltransferases and/or demethylases, Zhu et al. (2020) analyzed their transcript levels in peach fruit. The results of correlation analyses indicate that DNA methylation status in postharvest peach fruit might be maintained by the DNA methyltransferase genes *PpDRM1* and *PpDRM2* as well as the DNA demethylase gene *PpDML3*. Rothkegel et al. (2021) reported that the expression level of DNA methyltransferases *PpeMET1*, *PpeCMT3*, *PpeDRM2*, and *PpeDMR2.2* decreased after fruit CS. In addition, they observed that DNA demethylases *ROS1*, *DME*, and *DML2* were downregulated in damaged fruits after CS. These findings indicate that methyltransferases and demethylases expression could regulate cold-induced DNA methylation levels in *Prunus* fruits. CI-affected fruits exhibit higher methylation levels in comparison to CI-free fruits, indicating that CI-free fruits have better DNA methylation regulation via differential expression of methyltransferases and demethylases (Zhu et al., 2020; Rothkegel et al., 2021; Zhu et al., 2021). So far, no information about CI regulation mediated by histone post-transcriptional modifications (HPTMs) or chromatin accessibility was found in the literature.

Rothkegel et al. (2021) detected a variation in the methylation level between chromosomes when comparing normal and mealy fruits. These methylation differences co-localized with previously reported QTLs in chromosomes 4 and 5 for mealiness (Cantín et al., 2010; Nuñez-Lillo et al., 2015; Nuñez-Lillo et al., 2019). Candidate genes within these QTLs that presented differentially methylated regions (DMRs) between mealy and normal fruits at harvest were related to an increase in gibberellin biosynthesis, signal transduction, protein transport, and a decrease in cell wall modifications. Rothkegel et al. (2021) integrated the annotation of DMRs and differentially expressed genes (DEGs) after fruit storage and identified a *CYP450 82A* and an UDP-ARABINOSE 4 EPIMERASE 1 genes that were downregulated and hypermethylated in mealy fruits, which might affect iron homeostasis and cell wall metabolism of stone fruits, respectively. Intriguingly, these genes co-localized with transposable elements (TEs) suggesting that the observed differences in DNA methylation could be related to the presence of these TE and to consequent genetic variation between genotypes (Rothkegel et al., 2021). More in details, the authors suggested that the presence of TEs can affect the DNA methylation in non-coding regions and the differences in TEs between siblings may cause dynamic methylation profiles between individuals. While comparing mealy and normal fruits at harvest, Rothkegel et al. (2021) found that mealy fruits showed hypermethylation of the CHH context, therefore, they hypothesized that the genetic variance between siblings results in increased methylation in CI susceptible fruits. Therefore, the methylation status of a fruit at harvest could be indicative of the fruit’s responses to CS and CI development.

Rothkegel et al. (2021) also detected differences in DNA methylation in genomic regions and methylation contexts, with higher methylation levels in CG context in transcribed regions in comparison to CHG and CHH contexts. In addition, Zhu et al. (2021), reported that DNA methylation changes in different genomic regions of CI-related genes are associated with their transcript levels. Whole genome methylation was negatively correlated in upstream and downstream regions with gene expression, but the correlation was positive for the gene body. Zhu et al. (2021) explain that since there is a complex relationship between gene expression and methylation level in different regions of the gene, the association between DNA methylation and gene expression or repression depends on genome regions and methylation context. For example, ethylene-related genes *PpSAM1/2*, *PpACO1/2*, *PpETR2*, and *PpEIN3* showed a negative correlation between gene expression and DNA methylation levels in gene promotor and/or upstream regions; while *PpACS2*, *PpETR1*, and *PpERS1* showed a positive correlation. Similar observations were made for genes related to softening, IB, and ROS metabolism.

Multi-omics studies (Monti et al., 2019; Brizzolara et al., 2020) have revealed how differences in transcripts, proteins and metabolites can affect CI incidence between cultivars, and how the same cultivars can produce normal and CI phenotypes with different degrees of severity. Brizzolara et al. (2020) determined that LOX activity, responsible for the production of volatiles (Zhang et al., 2022), and LOX-associated volatiles varied between genotypes and applied stress, although no clear relationship to CI incidence has been provided. However, this study suggested that different expressions of temperature-responsive *CBF* genes (*PpCBF1* and *PpCBF6*) are associated with different CI development in Flaminia and Redhaven varieties. *CBF* expression levels varied between storage temperature and timing, indicating that genotypes have differences in regulatory mechanisms. Differences in phenotypes within the same variety can be related to content differences in protective mechanisms against cold stress (Monti et al., 2019). The Spring Lady variety can produce contrasting woolly phenotypes, where woolly phenotypes showed a significant decrease in sugars, sugar alcohols, organic acids, and amino acids in comparison to healthy phenotypes. Woolliness development was linked to sugar catabolism, amino acid usage, and proteome reconfiguration. Monti et al. (2019) hypothesized that the redox status of the peach fruit might be the initial signal to trigger the metabolic and proteomic differences associated with woolly phenotypes.

#### 2.2.2 CI mitigation strategies in *Prunus* fruits

Despite multiple CI alleviation treatments in stone fruits (Rodrigues et al., 2022) not all treatments have been studied from a multi-omics point of view. Multi-omic approaches have been used to study CI alleviation, including hot water (HW), melatonin, methyl jasmonates (MeJa), jasmonic acid (JA), salicylic acid (SA), low-temperature conditioning (LTC) and pre-conditioning (PC) treatments. These complementary treatments to CS alter biochemical and molecular mechanisms that alleviate CI.

Several multi-omics approaches suggest that the regulation of the ethylene pathway is involved in CI alleviation (Tanou et al., 2017; Wang et al., 2017; Zhu et al., 2019; Duan et al., 2022). Wang et al. (2017) and Zhu et al. (2019) highlight the role of endogenous ethylene in CI mitigation by using LTC and exogenous ethylene application, respectively. Both studies suggest a potential correlation between ethylene-responsive factors (ERFs) and genes involved in cell wall and lipid metabolism, which allow normal softening and reduce BI after storage. Although both treatments increased endogenous ethylene production, different mechanisms to mitigate CI have been proposed. Both studies point out the role of phospholipid acids in membrane stability, however, LTC increased membrane stability was related to increased monounsaturated and polyunsaturated fatty acids and sphingolipids contents, with an up-regulation of glucosylceramide biosynthesis, while exogenous ethylene promotes glycerolipids biosynthesis and ceramide catabolism.

Ethylene inhibition by melatonin application can alleviate CI in climacteric plums after CS (Xu et al., 2022). The accelerated production of ethylene after storage is associated with flesh reddening (Wang et al., 2016). Xu et al. (2022) demonstrated that melatonin inhibited the upregulation of the main components in the ethylene signaling transduction pathway which was accompanied by a reduced anthocyanins content, related to flesh reddening in plums. This idea is further supported as melatonin lowered the contents of secondary metabolites by suppressing genes involved in anthocyanin biosynthesis and MYB transcription factors which are associated with reddening index, anthocyanin content and cyanidin-3-O-glucoside (Xu et al., 2022). Melatonin-mediated ethylene inhibition is widely documented in fruit postharvest, but the reduction in anthocyanin content might be contradictory (Li et al., 2023). In plum, melatonin treatments have increased (Arabia et al., 2022) and reduced (Cortés-Montaña et al., 2023) anthocyanin content in the mesocarp. Xu et al. (2022) argue that melatonin regulation on fruit biochemical metabolism and physiological progress might vary between and within species [e.g. between climacteric and suppressed climacteric plum cultivars, Candan et al. (2011)] and concentration applied. Additionally, the application timing, number of applications, and cultivar specific responses might be involved in these contrasting behaviors.

Alterations in the softening capacity are a main effect of CS in stone fruits (Lurie and Crisosto, 2005). Interestingly, the increase of endogenous ethylene by LTC and exogenous ethylene was related to an enhancement in the softening capacity in peaches after CS. Both studies explain that endogenous ethylene upregulates the cell wall metabolism in peaches (Wang et al., 2017; Zhu et al., 2019). Contrarily, the reduction of ethylene burst in plums by melatonin was accompanied by a downregulation of genes encoding enzymes related to cell wall disassembly, mainly polygalacturonase. This suppressed the rapid decline in firmness, however, the treatment was not effective at maintaining fruit texture (Xu et al., 2022). Ethylene inhibition also caused a delay in the postharvest firmness loss in tomato, and a general delay in ripening, by using heat treatments (Pu et al., 2020).

Temperature treatments in stone fruits applied before CS have been proven to be involved in CI mitigation. This has been achieved by using HW submersion at 45°C for 10 minutes (Wang L. et al., 2021) and PC at 20°C for 48 h (Tanou et al., 2017), however, the mechanism involved are different between the two treatments. The pathways of the differential metabolites in HW treatments were associated with amino acids and phenolic metabolisms, which were strongly related to an enhanced antioxidative capacity, particularly, metabolites involved in arginine and proline metabolism, and phenylpropanoid and flavonoid biosynthesis. PC-treated fruits had a higher concentration of several amino acids before storage. Amino acid accumulation, particularly valine and isoleucine, was suggested as a priming strategy for CI alleviation. The upregulation of two genes (*ALS* and *KARI*) involved in the superpathway of valine, isoleucine, and leucine biosynthesis further supported this idea. Major changes in metabolites occurred during the CI development phase, therefore, this suggests fruits undergo a metabolic reconfiguration during the chilling period and the phenotypic response is dependent on the pre-chilling history of the fruits (Tanou et al., 2017). When applied in tomatoes, heat treatment induced DNA methylation changes of the CpG island of genes involved in ethylene biosynthesis and signaling resulting in important changes in the postharvest life dynamics of heat-treated fruit (Pu et al., 2020). The available evidence suggests that heat treatments could induce DNA methylation also in stone fruit and opens the possibility of using heat to control postharvest ripening and mitigate CIs. However, additional research is needed to fully understand and confirm these findings.

MeJA was effective at reducing BI in peaches, allowing a normal softening, promoting ethylene production, and increasing aroma volatiles (Duan et al., 2022). During the shelf-life evaluation, gene ontology (GO) revealed that genes associated with JA metabolic process, cell wall macromolecule catabolic process, lipid catabolic process, and DNA methylation were significantly enriched. MeJA treatments induced the accumulation of endogenous JA and Jasmonoyl-isoleucine (JA-Ile) by upregulating JA biosynthesis genes. Exogenous JA treatments were also effective at reducing CI by altering the ethylene biosynthetic pathway (specifically *ACS1*) and reducing the expression of IB-related genes (i. e. *PPO2* and *POD2*) (Zhao et al., 2021a; Zhao et al., 2021b). Even though MeJa induces endogenous JA, exogenous JA treatments might alleviate CI by an autonomous mechanism. MeJa treatments were able to maintain cell wall metabolism after storage by upregulating polygalacturonase, pectate lyase, and expansin-related genes. Additionally, MeJA treatment can regulate the synthesis of aroma volatiles as esters, lactones, and apocarotenoids, as genes related to their synthesis (*PpLOX1*, *PpHPL1*, *PpADH1*, *PpATT1*, and *PpCDD4*) are maintained during shelf-life, further enhancing fruits quality and acceptance. Duan et al. (2022) correlated the methylation levels with genes related to CI development after CS. DNA hypomethylation was associated with increased expression of genes related to ethylene biosynthesis (*ACS1*), fruit ripening (*NAC1*), fruit softening (*EXP1*), and volatile synthesis (*AAT1*). The authors suggest MeJA-induced DNA methylation is involved in gene expression and inhibition, which causes a transcriptional reprogramming that prevents CI development.

Similarly to JA, SA resulted in higher H_2_O_2_ content by repressing POX and catalase, enzymes related to H_2_O_2_ degradation. Both treatments promoted sucrose accumulation through different regulation of the expression of genes involved in sucrose metabolism and several metabolomic approaches have emphasized sugar accumulation in CI-free phenotypes (Lauxmann et al., 2014; Tanou et al., 2017; Monti et al., 2019; Zhao et al., 2021b). However, the underlying mechanism of SA and sucrose capability in the alleviation of peach CIs is still unclear (Zhao et al., 2021b). As mentioned by Tanou et al. (2017) sugar accumulation could be casually related to cold acclimatation or they could indirectly serve as energy or carbon source for further metabolic changes related to an enhanced CI acclimatation, as previously reported in Arabidopsis (Klotke et al., 2004). The latter hypothesis seem more supported because energy metabolism is usually deficient in CI-affected fruits (Xu et al., 2022). Additionally, sugars could enhance the antioxidant capacity, stabilize cell membrane structure and serve as osmoprotectants (Lurie, 2022). Tanou et al. (2017) highlighted the modulations of proteins related to energy before CS and during ripening after CS, further suggesting the importance of energy during cold stress and CI alleviation. Melatonin promoted energy metabolism by stimulating endogenous ATP generation and, therefore, increased the adenylate energy charge (Xu et al., 2022). The application of glycine betaine (Shan et al., 2016), oxalic acid (Jin et al., 2014) and MeJA (Jin et al., 2013) treatments have also improved the energy status of peach fruits and improved chilling alleviation. Shan et al. (2022) reviewed the role of ATP in CI mitigation. Intracellular ATP helps CI alleviation by supplying energy for the fruit’s cellular metabolism, and additionally enhancing the membrane stability and the cell’s antioxidative capacity; while the generation of extracellular ATP, a damage-associated signal molecule, creates an imbalance in the defense system associated with membrane damage, ROS accumulation, energy shortage, and cellular dysfunction (Shan et al., 2022). Therefore, CI mitigation strategies maintain ATP homeostasis in fruits (Shan et al., 2022).

**Figure 2:**
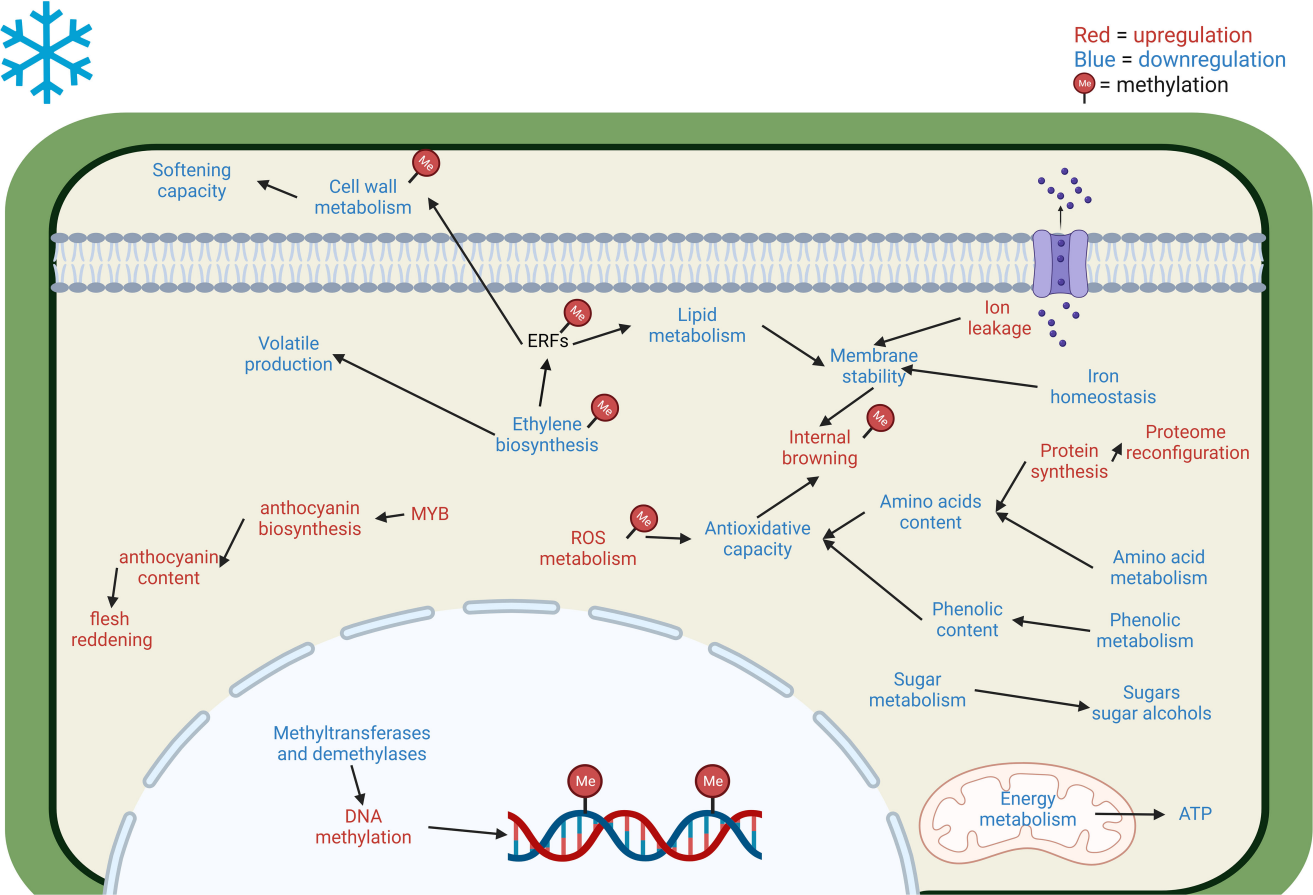
Main cellular and metabolic responses in Prunus fruits to cold stress revealed through multi-omics approaches. The blue letter coloring implies a downregulation of the pathway/decrease in the accumulation of the compound. On the other hand, red letter coloring represents an upregulation/increase in the accumulation of the compound. CI development is associated with reduced ethylene biosynthesis, which further affects the cell wall and lipid metabolisms via the ERF signaling pathway (Wang et al., 2017; Zhu et al., 2019). Cold-induced ROS imbalance results in ROS accumulation which is regulated by DNA methylation (Monti et al., 2019; Zhu et al., 2021). Ethylene inhibition negatively affects volatile content by reducing proteins involved in their production (Tanou et al., 2017; Lurie, 2022). High amino acid and phenolic content are related to CI tolerance by enhancing the antioxidative capacity of fruits, CI fruits have lower phenolic metabolism and amino acid metabolism (Tanou et al., 2017; Wang L. et al., 2021). Lower amino acid content may be related to increased protein synthesis leading to a proteome reconfiguration in CI-affected fruits (Monti et al., 2019). CI development is associated with iron deficiency (Rothkegel et al., 2021) and increased ion leakage which negatively affect membrane stability (Wang L. et al., 2021). Energy deficit is usual in CI-affected fruits and is associated with the downregulation of energy metabolism enzymes and ATP generation (Xu et al., 2022). Decrease in sugars and sugar alcohols are associated with CI development (Tanou et al., 2017; Monti et al., 2019), their specific role is unclear but could be related to energy source, antioxidative capacity, cell membrane stability and osmoprotection (Lurie, 2022). Cold-induced flesh reddening is associated with anthocyanin content, especially cyanidin-3-O-glucoside, the upregulation of anthocyanin biosynthesis is noted with higher expression of MYB transcription factors (Xu et al., 2022). Cold-induced DNA methylation changes are regulated by methyltransferases and demethylases, CI fruits have high methylation levels due to their lower control of DNA methylation (Rothkegel et al., 2021; Zhu et al., 2021); genes related to ethylene biosynthesis and signaling, cell wall metabolism, IB, and ROS metabolism are regulated by cold induced DNA methylation (Zhu et al., 2021).

Based on the multi-omics approaches evaluated, the main responses of stone fruits toward CS and CI development are presented in **Figure 2**. following the blueprint proposed by Albornoz et al. (2022). This model presents a general overview of CI induction and development in stone fruits; however, species and variety specific responses may vary and exhibit contrasting behavior.

All multi-omics studies presented here involve complementary treatments applied before CS, however, CI mitigation treatments can be applied during CS. A transcriptomic approach involving CA applied during CS was able to further reduce metabolic processes that CS alone and was effective at alleviation mealiness in nectarines (Sanhueza et al., 2015). The authors argue that by applying stresses together, cells respond hierarchically, and suggest that CA was the most important stress condition. It was previously known that CA reduces the respiration rate of fruits (Kader et al., 1989), additionally, Sanhueza et al. (2015) suggest changes affecting the primary metabolism. Further multi-omics approaches involving epigenomics could unveil the hierarchical responses to numerous stresses during fruit storage and provide a better understanding of CI mitigation.

### 2.3 Data integration

Next-generation sequencing technology (NGS) and the reduction of sequencing costs have allowed to sequence and annotate many plant genomes, including those of non-model species and crops of interest (Gapper et al., 2014). In Rosaceae, several annotated genomes of fruit crops have become available and can be accessed at the Genome Database for Rosaceae (GDR, https://www.rosaceae.org) (Jung et al., 2019). High throughput technologies have also facilitated the generation of extensive omics datasets (Mora-Poblete et al., 2023) eventually, increasing the number of studies involving omics data in fruit postharvest (Belay and James Caleb, 2022). Despite all these technological advances, some key aspects of fruits postharvest processes are not fully understood, including CIs (Sirangelo et al., 2022).

As reviewed by Amer and Baidoo (2021), integrated multi-omics approaches can provide a comprehensive understanding of biological systems and permit the identification of biomarkers with high accuracy, however, some drawbacks were also underlined. Multi-omics approaches are still expensive, deal with higher data volume and complexity, require a complex experimental design considering individual and combined omic technologies, and advanced data integration tools. Data integration is a bottleneck in the data analysis flowchart as it deals with heterogeneous data from multi-layer omics and tools are still being developed (Sirangelo et al., 2022). As we have previously discussed, multi-omics approaches have been used to elucidate CI development and mitigation in stone and pome fruits, however, data analysis and data integration approaches vary. Belay and James Caleb (2022) reviewed the use of integrated omics in fruit stress and defense response during postharvest and pointed out that most studies limit data integration to a linear data analysis approach. To overcome this limitation, the use of multivariate analysis and machine learning approaches are proposed as a viable solution (Belay and James Caleb, 2022). As mentioned by Scossa et al. (2021), crop improvement requires multi-omics approaches to train predictive and reliable models for important agronomic identification and exploitation. For CI research, machine-learning approaches have been used for CI detection in avocado (Guo et al., 2023) and cucumber (Lu and Lu, 2021). Other than CI detection, machine-learning approaches should be used to unravel the molecular mechanisms involved in CI development and alleviation. ChromHMM an unsupervised machine learning algorithm used to identify chromatin states, to annotate their occurrence across the genome, and facilitate their biological interpretation (Ernst and Kellis, 2017) has been used for CI research in bananas (Zhu et al., 2023). This study provided a multi-omics integrative approach to chromatin accessibility, histone modifications, distal cis-regulatory elements localization, transcription factor binding, and gene expression in cold-induced banana peel browning. The authors observed that cold-induced gene expression was generally associated with chromatin accessibility and enrichment of active histone marks along their promoters. Their results indicate that cold stress induces transcriptional reprogramming in browning pathways such as cold tolerance, phospholipid degradation and oxidation. Additionally, they observed that most browning-related genes were regulated via *WRKY* transcription factors by mediating enhancer-promoter interactions. This data integration approach highlighted the role of chromatin and histone modifications in CI gene transcriptional regulation.

Chromatin accessibility and histone modifications play a significant role in plant abiotic stress responses (Pandey et al., 2016; Halder et al., 2022; Nunez-Vazquez et al., 2022). Integrating chromatin regions accessibility and histone mark distribution permit to identify the location of genomic regulatory elements and understanding transcriptional regulation. Chromatin accessibility regions can be analyzed by combining enzymatic digestion of nuclear DNA and NGS, including DNase I Hypersensitivity sequencing (DNase-seq) Microccocal nuclease sequencing (MNase-seq), Assay for Targeting Accessible-Chromatin with sequencing (ATAC-seq) (Wang F. X. et al., 2021). Briefly, DNase-seq and MNase-seq use enzymes to cut DNA double strands and the sequencing result displays accessible regions of chromatin, while ATAC-seq detects the regions bound by transcription factors or occupied by nucleosomes.

Till now ATAC-Seq has been used only in model species, however the development of efficient procedures for extracting nuclei from crop plant cells will allow its wider application. As mentioned by Zhao et al. (2020), one of the major limitations to study epigenomics in plants it the low efficiency of chromatin extraction and ChIP-Seqprocedure in particular in non-model plants. Recently, Canton et al. (2022) developed a protocol for chromatin immunoprecipitation to study histone modifications in peach reproductive tissues. They remarked that for fruit mesocarp, the efficiency depends on the fruit developmental stage, being more efficient in the pre-ripening stage, as during the ripening stage the concentration of polysaccharides and other secondary metabolites significantly reduce extraction yield and quality. Efficient methods for epigenomic studies in fruit tissues are required to fully understand CI development. Despite the limitations, epigenomic studies in plants have found that plant epigenetic transcriptional regulation involves chromatin states or different histone modification dynamics acting sequentially or in combination, rather than a histone modification alone (Pan et al., 2017; Zheng et al., 2019); and that DNA methylation cross-talks with histone modification to define epigenetic landscapes (Zhao et al., 2022). No information regarding chromatin accessibility and HPTMs was found on CI mitigation for pome and stone fruits. Due to the evidence of complex epigenetic regulation of gene expression in plant stress responses, we suggest further CI research in fruits should couple transcriptomic and epigenomic data integration.

## 3 Future perspectives

CIs are the main limitation in stone and pome fruit storage, resulting in quality reduction and major economic losses. Despite the vast use of omics data to study CIs in stone and pome fruits, the analysis of each omics layer independently has proven inefficient to explain this complex trait. Multi-omics approaches have the potential to decipher the molecular mechanism in CI development and develop strategies for CI alleviation, however, data integration is still a major issue. It is expected that advances in bioinformatic tools will facilitate data integration, especially focusing on multivariate analysis and machine learning approaches.

As we have reviewed, many multi-omics studies in pome and stone fruit CI have created CI development models involving candidate genes, however, further research is required to validate them to further apply them in breeding programs. Alleviation strategies are cultivar-dependent and environmentally affected, which further adds complexity to establishing a general strategy. Several of the CI alleviation treatments include priming and acclimatation which stimulate the fruit’s memory, which refers to metabolic or epigenetic adjustments that influence the fruit’s behavior for future events (Reissig et al., 2023). CI alleviation treatments induce a metabolic reconfiguration by increasing cryoprotective molecules (Lurie, 2022) and induce DNA methylation changes associated with transcriptional reprogramming (Duan et al., 2022). In the case of CA, Sanhueza et al. (2015) propose a hierarchical behavior of fruits toward different stimuli applied simultaneously. Multi-omics studies can provide a broader understanding of fruit memory and the hierarchical responses of fruits to simultaneous stresses. Overall, multi-omics CI alleviation research should focus on optimizing cultivar dependent-strategies and developing CI tolerant cultivars.

Finally, the identification of potential biomarkers to discriminate chilling-injured from sound fruit at early stages of development of the disorder could help to optimize CS and fruit handling practices resulting in decreasing loss and waste. Based on multi-omics evidence reported in this review, candidate postharvest biomarkers for CS responses in pome and stone fruits are: ethylene production; sugar, amino acid, phenolic, lipid, aromatic volatile, and ATP contents; and the redox status. Among these, the most promising biomarkers are those showing an epigenetic regulation. It has been noted, in the literature, CIs are varying from orchard to orchard, indicating an environmental control of these disorders (Kahlaoui et al., 2022), in which epigenetic mechanisms can play a central role (Gapper et al., 2013).

## Author contributions

MR: Data curation, Writing – original draft. EO: Data curation, Writing – original draft. AR: Writing – review & editing. SV: Data curation, Supervision, Writing – review & editing. BR: Conceptualization, Supervision, Writing – review & editing. CB: Conceptualization, Funding acquisition, Supervision, Writing – review & editing.
